# Patterns and Predictors of Heroin Use, Remission, and Psychiatric Health Among People with Heroin Dependence: Key Findings from the 18–20-Year Follow-Up of the Australian Treatment Outcome Study (ATOS)

**DOI:** 10.1007/s11469-022-01006-6

**Published:** 2023-01-18

**Authors:** Christina Marel, Jack Wilson, Shane Darke, Joanne Ross, Tim Slade, Paul S. Haber, Katherine Haasnoot, Rachel Visontay, Madeleine Keaveny, Chris Tremonti, Katherine L. Mills, Maree Teesson

**Affiliations:** 1 University of Sydney Matilda Centre for Research in Mental Health and Substance Use, Sydney, NSW Australia; 2 National Drug and Alcohol Research Centre, UNSW Australia, Kensington, NSW Australia; 3 University of Sydney Addiction Medicine, Royal Prince Alfred Hospital, Camperdown, NSW Australia; 4 Sydney Local Health District Drug Health Services, Camperdown, NSW Australia

**Keywords:** Heroin dependence, Psychiatric comorbidity, Overdose, Cohort, Mortality, Longitudinal

## Abstract

This study aimed to investigate the long-term patterns and predictors of heroin use, dependence, and psychiatric health over 18–20 years among a cohort of Australians with heroin dependence, using a prospective longitudinal cohort study conducted in Sydney, Australia. The original cohort consisted of 615 participants, who were followed up at 3 months and 1, 2, 3, 11, and 18–20 years post-baseline; 401 (65.2%) were re-interviewed at 18–20 years. The Australian Treatment Outcome Study structured interview with established psychometric properties was administered to participants at each follow-up, addressing demographics, treatment and drug use history, overdose, crime, and physical and mental health. Overall, 96.7% completed at least one follow-up interview. At 18–20 years, 109 participants (17.7%) were deceased. Past-month heroin use decreased significantly over the study period (from 98.7 to 24.4%), with one in four using heroin at 18–20 years. Just under half were receiving treatment. Reductions in heroin use were accompanied by reductions in heroin dependence, other substance use, needle sharing, injection-related health, overdose, crime, and improvements in general physical and mental health. Major depression and borderline personality disorder (BPD) were consistently associated with poorer outcome. At 18–20 years, there is strong evidence that clinically significant levels of improvement can be maintained over the long term. The mortality rate over 18–20 years was devastating, with over one in six participants deceased. More sustained and targeted efforts are needed in relation to major depression and BPD to ensure evidence-based treatments are delivered to people with heroin dependence.

**Supplementary Information:**

The online version contains supplementary material available at 10.1007/s11469-022-01006-6.

Heroin use is a significant public health concern internationally. While substantial international research and public health investment have been made over the past few decades to address the opioid crisis, opioid-related deaths have dramatically increased, doubling in both the USA and Scotland over the past 10 years (van Amsterdam et al., 2021; CDC WONDER, 2020, 2021; Pierce et al., 2021). The burden associated with heroin dependence is higher in Australia compared with any other country, with the Australian rate of years of life lived with disability per 100,000 is 196.8, a rate that is close to twice that of the entire global population (104.3 per 100,000) (Degenhardt et al., 2014). In Australia, one in four drug-induced deaths in 2019 was due to heroin, the highest proportion since 1997 (Chrzanowska et al., 2021), and deaths related to opioid use disorders have increased by 41% over the same period (UNODC, 2021; Vos et al., 2020). It is therefore not surprising that heroin dependence is associated with high levels of morbidity and mortality and a greater burden of disease than any other illicit drug class (Degenhardt et al., 2013, 2019; National Institute on Drug Abuse, 2021).

Research suggests, however, that a lifelong course of cycling in and out of treatment, periods of relapse, chronic poor health, and premature death is not inevitable; studies examining patterns of opioid use in longitudinal cohorts from the USA, UK, and Australia have typically identified a group of people who use at low levels throughout the study period (Eastwood et al., 2018; Hser et al., 2007, 2017; Teesson et al., 2017). While understanding the long-term patterns and predictors of heroin use, remission, and psychiatric health remains a critical health issue internationally, fundamental ideological differences in international approaches to opioid and heroin dependence make investigation of key outcomes important. For example, differences in national drug strategy policy (i.e. harm minimisation versus abstinence-based or ‘zero-tolerance’ approaches) include the availability of universal healthcare, ready access to subsidised prescription medications including pharmaceutical opioids and opioid pharmacotherapy, social structure/welfare support, needle-exchange programmes, and low prevalence of HIV and other blood-borne viruses (McBride et al., 2009; The National Centre for Education and Training on Addiction (NCETA), 2021). All these factors may have implications for treatment outcomes.

Longitudinal cohort studies remain the ideal method of investigating the natural history of heroin dependence. Limitations of research to date, however, include an almost exclusive focus on treatment entrants; a restricted range of outcome measures (largely limited to opioid use); high attrition rates; and a lack of long-term follow-up, mostly consisting of less than 10 years.

The 18–20-year follow-up of the Australian Treatment Outcome Study (ATOS) provides a unique opportunity to build on these findings to examine longer-term patterns, predictors, and risk factors of heroin use, remission, and psychiatric health (Marel et al., 2020). Data collected over the first 11 years of ATOS highlighted greater heterogeneity in patterns of heroin use and remission than previously identified (Teesson et al., 2017). One-fifth of the cohort consistently demonstrated a high probability of use over the study period, emphasising that for some, heroin dependence is a chronic, debilitating disorder requiring a long-term response. At the other end of the spectrum, one in six participants demonstrated a rapid decrease in heroin use over the first 3 years followed by maintained abstinence (Teesson et al., 2017).

Extending these findings to 18–20 years, when ATOS participants were in their late forties, a critical period of transition associated with increased risk and prevalence of developing chronic disease (Atella et al., 2019; Australian Bureau of Statistics (ABS), 2022), is critical for addressing gaps in our knowledge and understanding of the natural history of heroin dependence and the long-term course of illness. The current study examined a range of outcomes for ATOS participants 18–20 years after they first entered the study. Specifically, the aims of the current study were to:

1. Examine the patterns of substance use, psychiatric health, criminal involvement, and mental and physical health of ATOS participants over the 18–20-year study period.
2. Ascertain whether any demographic, substance use, or treatment factors were associated with outcome over the 18–20-year study period.

## Method

### Design

ATOS is a naturalistic prospective longitudinal cohort study of 615 people with heroin dependence, recruited to the study in 2001–2002 (Ross et al., 2005). Follow-up interviews were conducted at 3 months and 1, 2, 3, 11, and 18–20 years post-baseline. Pre-specified outcomes and objectives are detailed in the study protocol (Marel et al., 2020). Ethics approval for the 18–20-year follow-up was obtained from the Sydney Local Health District Royal Prince Alfred Zone (X18-0512 & HREC/18/RPAH/733).

### Participants

Of the 615 participants, 535 were recruited as they entered treatment for heroin dependence (201 entering maintenance therapies, 201 entering detoxification, 133 entering residential rehabilitation) from 19 treatment agencies in the greater Sydney region. Agencies represented the major treatment modalities available for heroin dependence in Australia and were selected randomly from within treatment modality and stratified by regional health area. A comparison group of 80 people who were not in treatment was recruited from needle and syringe programmes within the same regional health areas as treatment participants.

Follow-up interviews were conducted with 549 (89.3%), 495 (80.5%), 469 (76.3%), 429 (69.8%), 431 (70.1%), and 401 (65.2%) participants at 3 months and 1, 2, 3, 11, and 18–20 years, respectively (Supplementary Fig. 1; Ross et al., 2005; Teesson et al., 2015). The vast majority of the cohort (96.7%) completed at least one follow-up interview.

Baseline factors associated with retention in the study at 18–20 years were examined using a series of binomial logistic regressions, with variables hypothesised to have a potential impact on retention. These included index treatment modality, age, sex, number of heroin use days in the preceding month, number of drug types used in the preceding month, current major depression, current post-traumatic stress disorder (PTSD) symptomatology, antisocial personality disorder (ASPD), borderline personality disorder (BPD), past-month use of tobacco, other opiates, amphetamines, cocaine, hallucinogens, benzodiazepines, antidepressants, alcohol, cannabis, inhalants, past-month injection-related health problems, past-month unstable housing, and general mental and physical health scores. The only statistically significant predictor of loss to follow-up was older age (odds ratio (OR) 0.97; 95% confidence interval (CI): 0.95 to 0.99) indicating that the sample re-interviewed at 18–20 years was broadly representative of the initial sample of 615 enrolled in ATOS.

### Structured Interview

The baseline and follow-up interviews were administered utilising measures with established psychometric properties. Specific details of interview components have been previously described (Ross et al., 2005; Teesson et al., 2015). In brief, baseline interviews assessed demographic characteristics (including age, sex, main source of income, and accommodation in the past month), past-month heroin and other drug use (including other opiates, alcohol, cannabis, benzodiazepines, amphetamines, cocaine, hallucinogens, or inhalants), treatment history, injection-related health problems (past-month heroin-related overdose, abscess, or infections from injecting, whether the participant had experienced a ‘dirty hit’, prominent scarring, bruising or difficulty injecting), past-month criminal involvement, general physical and mental health, major depression, PTSD, ASPD, and BPD.

Past-month drug use and criminal involvement were assessed using the Opiate Treatment Index (OTI) (Darke et al., 1992). General physical health and mental health were measured using the Short-Form 12 (SF12) (Ware et al., 1996), in which lower scores indicate poorer health. The Composite International Diagnostic Interview version 2.1 (CIDI (World Health Organisation, 1993)) was used to assess DSM-IV diagnoses of past-month heroin dependence, major depression, lifetime trauma exposure, and PTSD. Participants were classified as having current PTSD if they met criteria for a lifetime diagnosis and had experienced symptoms in the preceding 12 months (Mills et al., 2007). DSM-IV diagnoses of ASPD were obtained with use of a modified version of the Diagnostic Interview Schedule (Robins et al., 1981), and the International Personality Disorders Examination Questionnaire was used to screen participants for ICD-10 BPD (Loranger et al., 1994).

Sections relating to demographics, drug use, injection-related health, criminal involvement, general physical and mental health, and depression were reassessed at each follow-up interview. Participants were also asked the number of times they had commenced treatment for heroin dependence since their last interview, the treatment type, and the duration of each episode (Teesson et al., 2015). To enhance participant recall, interviews were administered with use of the life chart approach (Hunt & Andrews, 1995), which ties interview questions to significant events in participants’ lives (e.g. births, deaths, marriages, divorces), and is based on the well-validated timeline follow-back (TLFB) method (Sobell & Sobell, 1992).

### Statistical Analyses

Categorical and continuous measures of outcome were analysed using a generalised estimating equations (GEE) approach, with an unstructured correlation matrix (Twisk, 2013). Categorical outcomes examined included past-month heroin use (yes/no), past-month overdose (yes/no), past-month heroin dependence (yes/no), past-month other substance use (yes/no), past-month needle sharing (yes/no), past-month injection-related health problems (yes/no), and past-month criminal involvement (yes/no). Continuous outcomes examined were past-month general physical health and past-month mental health.

GEEs account for the correlation among repeated measures within a longitudinal design and allow for the simultaneous examination of relationships between different variables at multiple time points. The estimated regression coefficients provided in the analysis reflect the relationship between the longitudinal development (i.e., change over time) of the outcome variable and the longitudinal development of corresponding predictor variables, as well as an estimate of the time-averaged difference between groups (Twisk, 2013). As GEEs use all available data, missing data were not imputed (Twisk & de Vente, 2002).

Fixed and time-varying covariates were included in the models. Fixed covariates (assessed at baseline) that were categorical included sex (male/female), ASPD (yes/no), BPD (yes/no), and current PTSD (yes/no). Age at baseline and years of education were the only continuous fixed covariates included. Categorical time-varying covariates (reassessed at each interview) included whether participants had received wage as a main source of income in the past month (yes/no), had entered maintenance therapy (yes/no), detoxification (yes/no) or residential rehabilitation (yes/no) since the last interview, and major depression (yes/no). The only continuous time-varying covariate that was included was the number of treatment episodes commenced between interviews. Significant results were reported as the estimate of the time-averaged difference between groups, and the longitudinal impact of a change in the independent variable on the odds or mean level of the dependent variable occurring (Twisk, 2013). All analyses were conducted using PASW Statistics 25.

## Results

### Baseline Cohort Characteristics

The characteristics of the cohort at baseline have been previously reported (Ross et al., 2005). In brief, the mean age of the cohort at baseline was 29.3 years (SD 7.8) and two-thirds (66.2%) were male. Participants had completed a mean of 10.0 years of school (SD 1.7), 29.1% had completed a trade/technical course, and 5.9% a university degree. Just under half (45.7%) reported government allowances as their main source of income in the past month, 23.9% reported criminal activity, and 17.6% indicated wage as their main source of income. Just over half (54.6%) reported past-month criminal involvement and 40.8% had spent time in prison. The mean duration of heroin use was 9.6 years (SD 7.4) and the mean number of drugs used in the past month was 9.0 (SD 1.7). There were high rates of psychopathology, with 24.6% meeting criteria for major depression, 41.1% for lifetime PTSD, 71.5% for ASPD, and 45.5% screening positively for BPD.

### Outcomes for Heroin Dependence over 18–20 Years

At 18–20 years, 109 participants were deceased (17.7%; 72 males, 37 female), which was a substantial increase from the 72 deaths observed at the 11-year follow-up (Darke et al., 2016). At baseline, 87% of participants were entering treatment for heroin dependence, 38% of whom were commencing maintenance therapies. At 18–20 years, 47.6% of the cohort were receiving treatment for heroin dependence, 35.9% of whom were receiving maintenance therapies. Over the follow-up period, all participants received treatment for their heroin dependence (Table 1), undertaking a median of six treatment episodes (range 1–92), over a median of 341.3 weeks (range 1–1034.6).

**Table 1:** Patterns of treatment exposure between interviews over the 18–20-year follow-up

	3 months	1 year	2 years	3 years	11 years	18–20 years
*% attending*						
Maintenance therapies^a^	44.9	56.6	58.6	57.5	74.2	64.1
Detoxification	37.9	18.2	13.0	8.6	38.8	18.5
Residential rehabilitation	30.1	19.8	13.9	7.7	36.1	17.7
Any treatment	91.4	80.6	72.0	65.2	88.1	68.1
Median no. treatment episodes (range)	1 (1–6)	1 (0–11)	0 (0–10)	0 (0–7)	0 (0–77)	1 (0–65)

^a^Maintenance therapies included methadone maintenance and buprenorphine

### Heroin Use

**Fig. 1:**
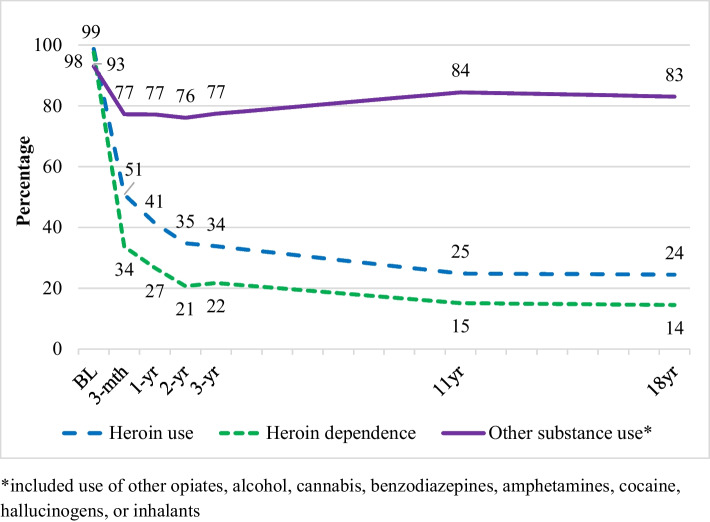
The prevalence of past-month heroin use, heroin dependence, and other drug use* across the 18–20-year follow-up period

The proportion of people who used heroin in the past month decreased significantly from baseline to 11 years (Fig. 1, Table 2) and remained stable to 18–20 years. Overall, the proportion of the cohort who were using heroin decreased from 98.7% at baseline to 24.4% at 18–20 years. Past-month abstinence from heroin was associated with having commenced residential rehabilitation (OR 1.64), while past-month heroin use was significantly associated with undergoing detoxification (OR 1.54) and major depression (OR 1.95). Neither time spent in maintenance therapies nor the number of treatment episodes was associated with past-month heroin use.

**Table 2:** General estimating equation results: substance use over time^a^

	Heroin use		Heroin dependence		Other substance use^b^	
	OR	95% CI	OR	95% CI	OR	95% CI
*Change over time*						
Baseline to 11 years	0.00***	0.00, 0.01	0.00***	0.00, 0.01	0.43**	0.23, 0.51
11 years to 18–20 years	1.02	0.76, 1.36	1.04	0.72, 1.51	0.79	0.55, 1.14
Baseline to 18–20 years	0.00***	0.00, 0.01	0.00***	0.00, 0.01	0.34***	0.23, 0.50
*Demographics*						
Male	1.05	0.82, 1.34	1.22	0.94, 1.58	1.57**	1.19, 2.09
Age	1.01	0.99, 1.02	1.00	0.99, 1.02	0.99	0.97, 1.01
Years of school completed	0.97	0.90, 1.04	0.99	0.92, 1.07	0.96	0.89, 1.05
Wage main source of income	0.83	0.61, 1.13	0.84	0.60, 1.18	0.97	0.68, 1.37
*Mental health*						
Major depression	1.95***	1.53, 2.47	2.18***	1.67, 2.85	1.64**	1.22, 2.21
BPD	1.10	0.86, 1.40	1.49**	1.17, 1.89	1.34*	1.01, 1.78
ASPD	0.99	0.76, 1.30	1.00	0.75, 1.34	1.15	0.84, 1.58
PTSD	0.82	0.64, 1.05	0.80	0.61, 1.03	0.89	0.66, 1.21
*Treatment*						
Maintenance therapy	1.19	0.98, 1.45	1.08	0.86, 1.37	1.66***	1.34, 2.05
Detoxification	1.54***	1.25, 1.90	1.94***	1.54, 2.46	1.28*	1.00, 1.64
Residential rehabilitation	0.61***	0.48, 0.77	0.59***	0.45, 0.77	0.37***	0.30, 0.46
Treatment episodes	1.02	0.99, 1.05	1.02	0.99, 1.06	0.97	0.95, 1.00

^***^p < 0.001

^**^p < .01

^*^p < 0.05

^a^ORs represent a pooled estimate of between and within-subject change over time

^b^Included use of other opiates, alcohol, cannabis, benzodiazepines, amphetamines, cocaine, hallucinogens, or inhalants

### Heroin Dependence

The proportion of people with heroin dependence decreased significantly from baseline to 11 years and remained stable to 18–20 years (Fig. 1, Table 2). The proportion who met criteria for heroin dependence decreased from 97.6% at baseline to 14.5% at 18–20 years. Heroin dependence was associated with having spent time in detoxification (OR 1.94), screened positive for BPD (OR 1.49), and met criteria for major depression (OR 2.18). Having spent time in residential rehabilitation was associated with a reduced odds of being heroin dependent (OR 0.59).

### Other Substance Use

The proportion of people who had used other substances in the preceding month decreased from baseline to 11 years and remained stable to 18–20 years (Fig. 1, Table 2). The rate observed at 18–20 years (83.0%) was significantly lower than the rate observed at baseline (93.0%). Other substance use was associated with having spent time in maintenance therapies (OR 1.66), having spent time in detoxification (OR 1.28), being male (OR 1.57), screening positive for BPD (OR 1.34), and the presence of major depression (OR 1.64). Having spent time in residential rehabilitation was associated with reduced likelihood of other substance use (OR 0.37).

### Injection-Related Health Problems

**Fig. 2:**
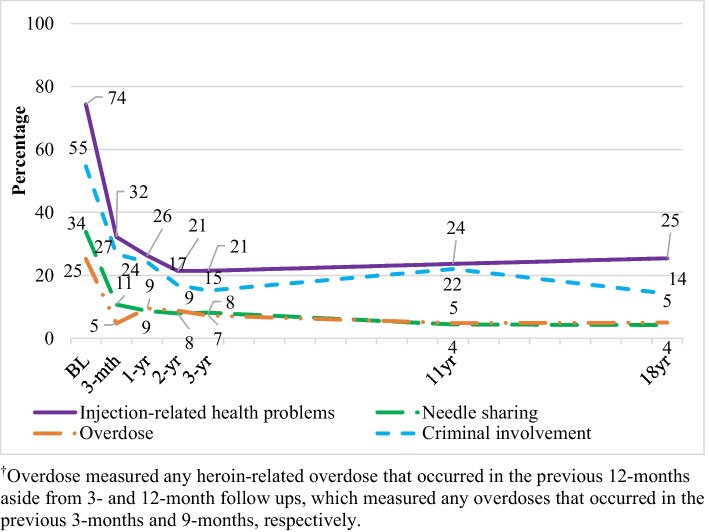
The prevalence of injection-related health problems, needle sharing, criminal involvement, and overdose^†^ across the 18–20-year follow-up period

The proportion of people with past-month injection-related health problems decreased from baseline to 11 years (Fig. 2, Table 3). While no observable change was evident between 11 and 18–20 years, the overall proportion of people with injection-related health problems decreased from 74.3% at baseline to 25.4% at 18–20 years. Injection-related health problems were associated with being female (OR 1.37), the presence of major depression (OR 2.04), ASPD (OR 1.49), screening positive for BPD (OR 1.54), and having spent time in detoxification (OR 1.26). Those who had spent time in residential rehabilitation were less likely to have experienced past-month injection-related health problems (OR 0.76).

**Table 3:** General estimating equation results: health problems and crime over time^a^

	Injection-related health problems		Overdose		Needle sharing		Criminal involvement	
	OR	95% CI	OR	95% CI	OR	95% CI	OR	95% CI
*Change over time*								
Baseline to 11 years	0.08***	0.06, 0.12	0.11***	0.05, 0.20	0.07***	0.04, 0.13	0.15***	0.11, 0.22
11 years to 18–20 years	1.13	0.83, 1.54	1.14	10.59, 2.21	0.99	0.48, 2.01	0.60**	0.41, 0.88
BL to 18–20 years	0.10***	0.07, 0.13	0.12***	0.06, 0.24	0.07***	0.04, 0.14	0.09***	0.06, 0.14
*Demographics*								
Male	0.73**	0.58, 0.92	1.06	0.77, 1.46	0.73*	0.55, 0.97	1.37*	1.07, 1.75
Age	1.01	1.00, 1.02	1.00	0.98, 1.02	0.98*	0.96, 1.00	0.96***	0.95, 0.98
Years of school completed	0.97	0.91, 1.04	0.95	0.86, 1.04	0.92*	0.84, 1.00	0.97	0.91, 1.04
Wage main source of income	0.87	0.64, 1.19	0.61*	0.41, 0.92	0.84*	0.58, 1.23	0.63*	0.45, 0.90
*Mental health*								
Major depression	2.04***	1.64, 2.53	1.19	0.88, 1.60	1.72***	1.29, 2.29	1.62***	1.30, 2.03
BPD	1.54***	1.23, 1.92	1.78***	1.30, 2.42	1.84***	1.36, 2.48	1.62***	1.29, 2.05
ASPD	1.49**	1.14, 1.94	1.52*	1.04, 2.22	0.97	0.71, 1.34	2.09***	1.58, 2.75
PTSD	0.91	0.72, 1.15	1.09	0.81, 1.48	0.84	0.63, 1.14	0.92	0.72, 1.18
*Treatment*								
Maintenance therapy	1.16	0.93, 1.43	0.53*	0.37, 0.76	0.86	0.62, 1.19	1.28*	1.02, 1.61
Detoxification	1.26*	1.00, 1.59	2.33*	1.63, 3.32	1.79**	1.25, 2.55	1.48**	1.17, 1.88
Residential rehabilitation	0.76*	0.59, 0.97	1.51*	1.06, 2.17	0.53**	0.33, 0.85	0.69**	0.53, 0.90
Treatment episodes	1.01	0.98, 1.04	1.05	1.00, 1.09	1.05**	1.02, 1.09	1.01	0.98, 1.04

^***^p < 0.001

^**^p < .01

^*^p < 0.05

^a^ORs represent a pooled estimate of between and within-subject change over time

### Overdose

The proportion of people who had overdosed in the 12 months prior to the interview decreased significantly from baseline to 11 years. While no observable change was evident between 11 and 18–20 years, the overall proportion of people who overdosed decreased from 25.2% at baseline to 5.0% at 18–20 years (Fig. 2, Table 3). Overdose was associated with having spent time in detoxification (OR 2.33) or residential rehabilitation (OR 1.51), screening positive for BPD (OR 1.78) or meeting criteria for a diagnosis of ASPD (OR 1.52). Those who had spent time in maintenance therapies and those who nominated wage as the main source of income at baseline were less likely to have overdosed in the previous 12 months (ORs 0.54 and 0.61 respectively).

### Needle Sharing

Past-month sharing of needles decreased from baseline to 11 years and remained stable to 18–20 years (Fig. 2, Table 3). The overall proportion of people who shared needles decreased from 33.8% at baseline to 4.3% at 18–20 years. Needle sharing was associated with having spent time spent in detoxification (OR 1.79) and a greater number of treatment episodes (OR 1.05). Those who screened positive for BPD were also more likely to share needles (OR 1.84), as were those who met criteria for major depression (OR 1.72). Being male (OR 0.73), being older aged (OR 0.98), having completed more years of education (OR 0.92), nominating wage as a main source of income (OR 0.84), and having spent time in residential rehabilitation (OR 0.53) were associated with reduced likelihood of needle sharing.

### Criminal Involvement

The proportion involved in criminal activity decreased from baseline to 11 years, and further declined from 11 to 18–20 years. As illustrated in Fig. 2 and Table 3, the overall proportion of participants who were involved in past-month criminal activity decreased from 54.6% at baseline to 14.2% at 18–20 years. Criminal activity in the preceding month was associated with not having commenced residential rehabilitation (OR 0.69), and with having spent time in detoxification (OR 1.48) or maintenance therapies (OR 1.30). Criminal involvement was also associated with being younger (OR 0.96), male (OR 1.37), not nominating wage as a main source of income (OR 0.63), the presence of major depression (OR 1.62), ASPD (OR 2.09), and screening positive for BPD (OR 1.62).

### General Physical Health

**Fig. 3:**
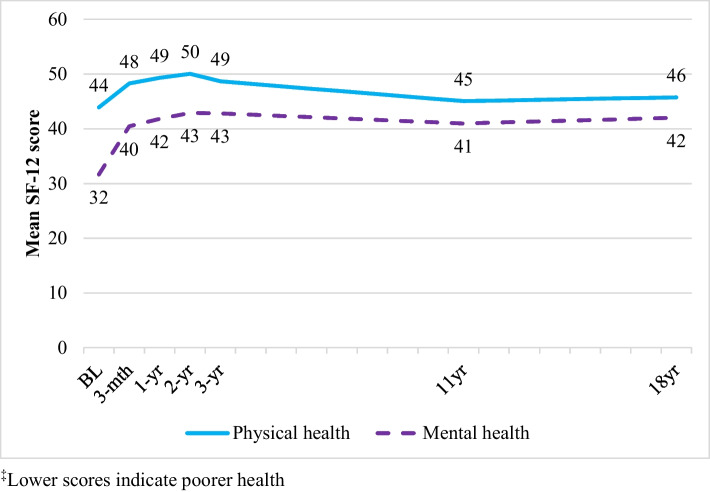
Mean SF-12 physical and mental health scores^‡^ across the 18–20-year follow-up period

As illustrated in Fig. 3 and Table 4, the physical health of the cohort improved significantly between baseline and 18–20 years; however, the difference was small (*β* = 1.72). Improved physical health was associated with having spent time in residential rehabilitation (*β* = 1.56), being male (*β* = 1.77) and having completed more years of education (*β* = 0.59). Poorer physical health was associated with having spent time in maintenance therapies (*β* =  − 0.95), being older (*β* =  − 0.21), the presence of PTSD (*β* =  − 1.23), and major depression (*β* =  − 2.10).

**Table 4:** General estimating equation results: physical and mental health over time^a^

	Physical health		Mental health	
	Regression coefficient (*β*)	95% CI	Regression coefficient (*β*)	95% CI
*Change over time*				
BL to 11 years	1.29	− 0.14, 2.71	10.06***	8.34, 11.18
11 years to 18–20 years	0.43	− 0.80, 1.67	0.30	− 1.07, 1.67
BL to 18–20 years	1.72*	0.36, 3.08	9.76***	8.34, 11.18
*Demographics*				
Male	1.56**	0.47, 2.64	1.24*	0.12, 2.36
Age	− 0.21***	− 0.28, − 0.14	− 0.09*	− 0.15, − 0.02
Years of school completed	0.59***	0.28, 0.90	0.14	− 0.19, 0.47
Wage main source of income	0.67	− 0.48, 1.81	0.66*	0.12, 2.36
*Mental health*				
Major depression	− 2.10***	− 3.14, − 1.06	− 12.25***	− 12.25, − 11.30
BPD	− 0.68	− 1.71, 0.36	− 3.77***	− 4.87, − 2.69
ASPD	− 0.25	− 1.41, 0.90	− 0.54	− 1.77, 0.68
PTSD	− 1.23*	− 2.35, − 0.12	− 0.74	− 1.88, 0.39
*Treatment*				
Maintenance therapy	− 0.95*	− 1.78, − 0.12	− 0.04	− 1.03, 0.94
Detoxification	− 0.66	− 1.53, 0.21	− 2.58***	− 3.70, − 1.46
Residential rehabilitation	1.56**	0.61, 2.51	0.33	− 0.76, 1.42
Treatment episodes	0.06	− 0.09, 0.22	− 0.06	− 0.25, 0.13

^***^p < 0.001

^**^p < .01

^*^p < 0.05

^a^Regression coefficients represent a pooled estimate of between and within-subject change over time

### General Mental Health

The general mental health of the cohort improved significantly from baseline to 11 years, and while no significant change was observed between 11 and 18–20 years, the overall general mental health of the cohort improved from baseline to 18–20 years (Fig. 3, Table 4). Improved mental health was associated with being male (*β* = 1.27) and nominating wage as a main source of income (*β* = 0.66), while poorer mental health was associated with having spent time in detoxification (*β* =  − 2.58), being older (*β* =  − 0.09), the presence of major depression (*β* =  − 12.25), and screening positive for BPD (*β* =  − 3.77).

## Discussion

By 18–20 years, there was considerable mortality with just over one in six participants deceased. While there was a substantial reduction in heroin use and dependence, one in four were currently using heroin, one in seven were heroin dependent, and just under half were in treatment. Concomitant improvements were found in relation to every other outcome examined, including reduced use of other substances; reductions in rates of needle sharing, injection-related health problems, overdose, and criminal involvement; and improvements in general physical and mental health. The pattern of results observed in relation to change over time was consistent with the findings at 11 years. With the exception of past-month criminal involvement (where further reductions were observed between 11 years to 18–20 years), improvements observed from baseline to 11 years were maintained throughout 18–20 years in relation to all outcomes. For most outcomes, the degree of improvement was substantial, but the majority of participants continued to use other substances. However, data were not collected that allows for a determination as to whether the use of those substances was at levels that would be indicative of problematic use. Nonetheless, these findings provide strong evidence that clinically significant levels of improvement can be maintained over the long term; remarkable given that upon entry to the study, the mean age of the cohort was 29.3 years (standard deviation [SD] 7.8) and participants had been using heroin for a mean of 9.6 years (SD = 7.4) (Ross et al., 2005, 2006).

Of considerable clinical interest are factors associated with treatment outcomes that can inform the planning and provision of services. Major depression was a robust and enduring predictor of poorer outcomes for almost every indicator examined over the 18–20 years, and had a stronger relationship with heroin use, heroin dependence, and poorer physical and mental health than any other variable examined. Effective treatments for major depression as a single disorder and in the presence of comorbid substance use disorders exist (Marel et al., 2022), but the persistent and pernicious nature of the relationship between major depression and poor outcomes for heroin dependence in the current study suggests that evidence-based treatments are not being provided to the most vulnerable when they need them.

Screening positive for BPD was similarly associated with lower levels of improvement in relation to the majority of outcomes. While this finding is consistent with the literature, to our knowledge, the current study is the only study to examine the impact of BPD over such a long period of time. While there are several treatment options available, dialectical behaviour therapy (DBT) modified for people with co-occurring BPD and substance use disorders (DBT-S) is the preferred treatment approach to date (Marel et al., 2022). Increased availability of evidence-based treatments for this comorbidity may play a critical role in improving outcomes among this group.

Unsurprisingly, ASPD was associated with criminal involvement, injection-related health problems, and overdose; PTSD on the other hand was associated only with poor physical health. The lack of association between PTSD and a broader range of outcomes, while encouraging, should be viewed with caution given that we operationalised PTSD as a lifetime diagnosis with symptoms experienced in the last 12 months in the current study, and may not be reflective of those with a current diagnosis of PTSD.

The relationship between treatment and long-term outcomes was not straightforward. Commencing residential rehabilitation was consistently associated with positive outcomes and was the only significant treatment factor related to improved general physical health. At 18–20 years, participants were in their late forties, a critical period associated with increased risk and prevalence of developing chronic disease (Atella et al., 2019; Australian Bureau of Statistics (ABS), 2022). The association between residential rehabilitation and improved general physical health in the current study may be reflective of the capacity of residential settings to provide a more holistic and comprehensive treatment approach, addressing the physical health of people with heroin dependence.

Notably, however, the inverse relationship was observed for overdose, where residential rehabilitation was consistently associated with increased odds of experiencing overdose across the 18–20-year study period. These findings concur with the broader literature which has highlighted the increased risk of overdose due to a reduction in tolerance following periods of abstinence, such as treatment and release from prison (Bell & Strang, 2020; Binswanger et al., 2013; Bukten et al., 2017; Darke, 2011; Davoli et al., 2007; Santo et al., 2021; Strang et al., 2003). Moreover, these findings point to the importance of providing psychoeducation regarding reverse tolerance and other harm reduction measures (e.g. take-home naloxone and training in its administration, supervised injecting sites) among people leaving residential rehabilitation and prison settings, in reducing opioid-related fatality (KPMG, 2010; Marshall et al., 2011; Razaghizad et al., 2021; Salom et al., 2021). These findings also have critical implications for policymakers when considering further funding of harm reduction schemes.

In contrast, but consistent with previous research (Bell and Strang, 2020; Darke, 2011; Santo et al., 2021), the current study found that maintenance therapy was consistently associated with reduced odds of overdose over the 18–20-year study period. As full or partial μ receptor agonists, methadone and buprenorphine effectively ‘block’ the ability of opioids to bind to opioid receptors, reducing the likelihood of overdose while a person receives maintenance therapies (Bell and Strang, 2020; Degenhardt et al., 2009). These findings are unique, however, in that much of the previous research has focused on fatal overdose, which is estimated to account for only a minority of all overdoses (Darke, 2011; Darke et al., 2003).

Consistent with international research (Chutuape et al., 2009; Gossop et al., 2000a), previous ATOS analyses indicated that although detoxification was associated with reductions in heroin use over the short term, it was related to poorer outcomes over the long term (Ross et al., 2006; Teesson et al., 2006, 2015). The consistent and enduring nature of the relationship between detoxification and poor outcome over 18–20 years provides further support for the important role it plays in providing an entryway into additional treatment, rather than standalone treatment.

ATOS is the first Australian cohort study to examine long-term outcomes among people with heroin dependence and provides a uniquely comprehensive analysis of the physical and psychiatric comorbidity experienced by this vulnerable group. Over 18–20 years, reductions were observed in relation to heroin use and dependence, other drug use, overdose, needle sharing, injection-related health problems, and criminal involvement. Despite these improvements, the mortality rate over 18–20 years was devastating, with just over one in six participants deceased: unacceptably high for those whose mean age was just 47 years.

As with all research, several limitations should be considered when interpreting the findings. Firstly, the study was primarily conducted with measures of self-report. While there is some contention about the use of self-report, there is international literature demonstrating its reliability and validity among people using heroin in research settings (Jackson et al., 2005; Napper et al., 2010). Secondly, while the process of recruitment involved random selection of treatment agencies stratified by modality, care should be taken in generalising results to treatment settings outside Australia. Demographic characteristics and drug use histories were consistent with previous international studies of people with heroin use (Darke et al., 2002; Gossop et al., 2000b; Hubbard et al., 1997). As with all longitudinal studies, there was loss to follow-up experienced in this study. However, the 65.2% follow-up rate after 18–20 years in such a complex population group, who were followed up during COVID-19, is remarkable.

While, ideally, naturalistic studies examining heroin dependence would commence prior to the onset of heroin use, to conduct such a study would require an initial longitudinal study of approximately 90,000 people (assuming 0.9% of the population are dependent), which is not feasible under current funding models. Future research should pursue further opportunities to conduct such studies.

Findings suggest residential rehabilitation may play a key role in improving the mental and physical health of people with heroin dependence, with the potential capacity to provide comprehensive and holistic treatment approaches to an ageing population. While rates of overdose declined over the study period, the importance of overdose prevention strategies for people with heroin dependence cannot be overlooked and point to the need for more comprehensive and assertive aftercare following treatment discharge and prison release. Peer workers may also play a vital role in harm reduction services, with a recent systematic review from the UK highlighting their importance in overdose prevention (Mercer et al., 2021). Crucially, major depression and BPD remained consistent and enduring factors associated with poor outcome, suggesting that more sustained and targeted efforts need to be made to ensure evidence-based treatments are being delivered to people with heroin dependence.

## Supplementary Information

Below is the link to the electronic supplementary material.

Supplementary file1 (PDF 84 KB)

## Data Availability

Data are currently unavailable for this study.
