# Supplementary figures and images for: Patterns and Predictors of Heroin Use, Remission, and Psychiatric Health Among People with Heroin Dependence: Key Findings from the 18–20-Year Follow-Up of the Australian Treatment Outcome Study (ATOS)
Source: Int J Ment Health Addict. 2023 Jan 18:1–18. Online ahead of print. doi: 10.1007/s11469-022-01006-6 (PMC9847452; doi:10.1007/s11469-022-01006-6)

**Supplementary Figure I: ATOS study flow**

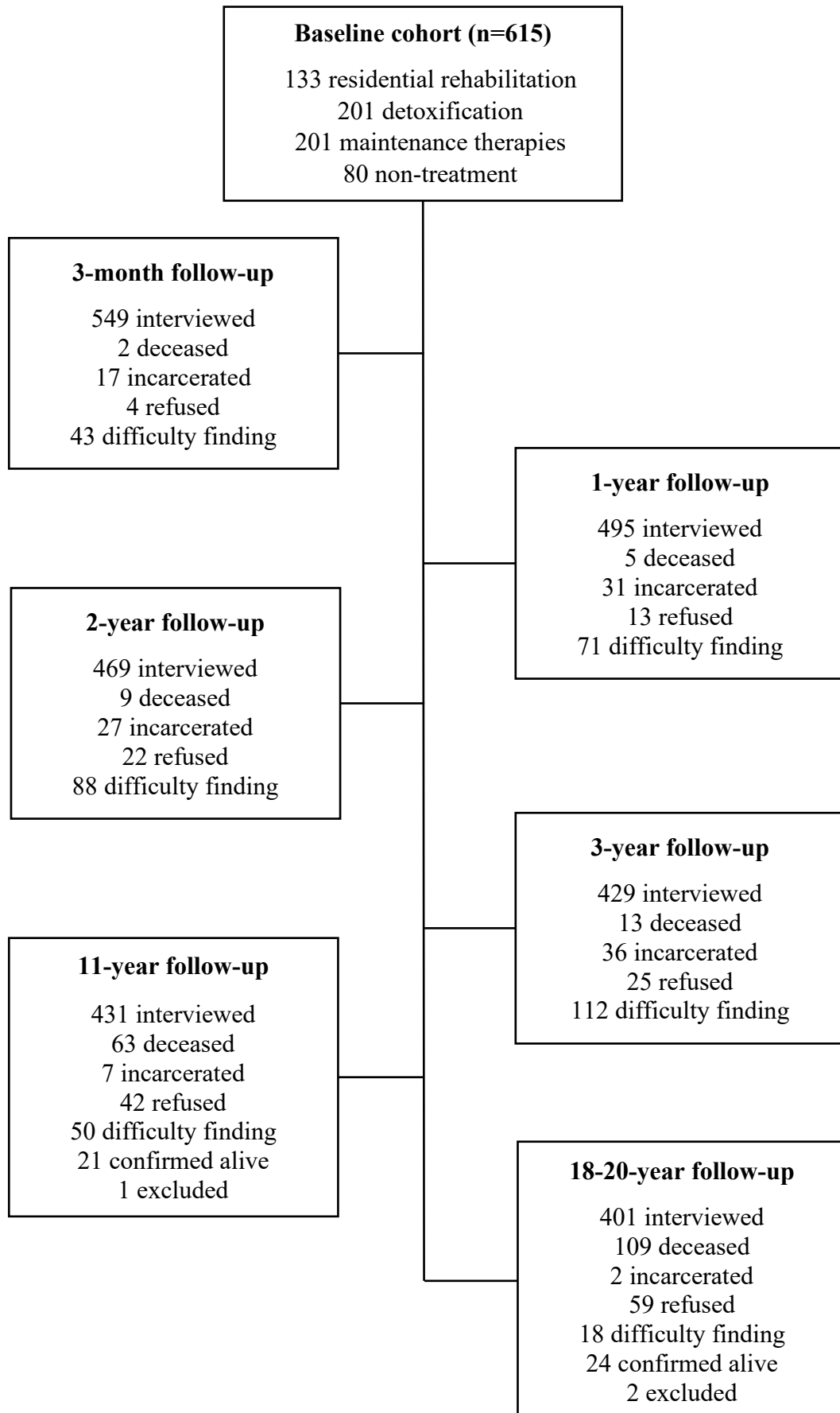

Supplement: Supplementary file 1 — Supplementary file1 (PDF 84 KB) [file 11469_2022_1006_MOESM1_ESM.pdf]
